# Study on the Influence of UV Light on Selective Antibacterial Activity of Silver Nanoparticle Synthesized Utilizing Protein/Polypeptide-Rich Aqueous Extract from The Common Walkingstick, *Diapheromera femorata*

**DOI:** 10.3390/ma17030713

**Published:** 2024-02-02

**Authors:** James Lee Cho, Luc Gaston Allain, Sanichiro Yoshida

**Affiliations:** Department of Chemistry and Physics, Southeastern Louisiana University, Hammond, LA 70402, USA; james.cho@selu.edu (J.L.C.); luc.allain@selu.edu (L.G.A.)

**Keywords:** silver nanoparticle, the common walkingstick, insect extract, green chemical synthesis, nano-biomaterials, UV-treated nanoparticles, UV exposure time-sensitivity

## Abstract

Common walkingstick (*Diapheromera femorata*) aqueous extract (CWSAE) can induce the synthesis of useful bionanomaterials. CWSAE is rich in water-soluble organic compounds such as proteins and polypeptides that function as reducing/stabilizing agents for nanoparticle formation from Ag^+^ ion precursors. The synthesized AgNPs exhibited a moderately uniform size, with the majority falling within the range of 20–80 nm. These AgNPs were UV-treated and tested as antibacterial agents to inhibit the growth of four pathogenic bacteria (*Burkholderia cenocepacia* K-56, *Klebsiella pneumoniae* ST258, *Pseudomonas aeruginosa* PAO1, and *Staphylococcus aureus* USA300), as well as one common bacterium (*Escherichia coli* BW25113). The disk diffusion test demonstrated that the UV-treated AgNPs significantly and selectively inhibited the growth of *Staphylococcus aureus* USA300 and *P. aeruginosa*, while showing a small effect on the other two species. This suggests the potential application of green-chemically synthesized AgNPs as selective antibacterial agents. Furthermore, we studied the effects of short-term (1–2 min) and long-term (5–30 min) UV treatment on the selective cytotoxicity of the AgNPs and found that the cytotoxicity of the AgNPs could depend on the duration of UV exposure against certain bacteria.

## 1. Introduction

Insect extracts contain various biomolecules such as proteins, peptides, and amino acids [1,2]. Some of these biomolecules may have reducing properties, allowing them to participate in the reduction of metal ions. These biomolecules can act as reducing agents, donating electrons to convert metal ions into nanoparticles [3]. In addition to proteins and biomolecules, insect extracts can contain organic compounds, including small molecules such as carbohydrates, alcohols, and organic acids [4]. Some of these compounds may have reducing capabilities and can contribute to the reduction process.

**Figure A1:**
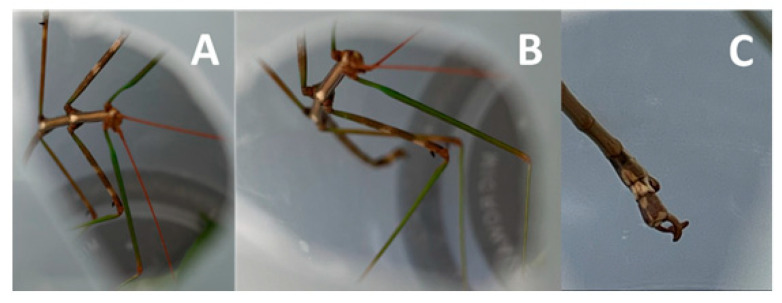
The common walkingstick (*Diapheromera femorata*): (**A**,**B**) Top view of The common walkingstick and (**C**) Close-up view of the each of the cerci of the common walkingstick.

The common walkingstick (CWS) (*Diapheromera femorata*) is one of the most widespread stick insects globally, including in the United States and Canada [5]. The body composition of CWS contains high levels of carbohydrates and proteins, yielding an aqueous extraction rich in water-soluble compounds and biological polymers. This includes hydrophilic polypeptides, carbohydrates, and DNA, as well as a small quantity of other compounds like polyphenols, flavonoids, and terpenoids [6]. A general description of CWS is provided in Appendix A (Figure A1).

Silver nanoparticles (AgNPs) are well-known for their significant antibacterial activity against many pathogenic bacteria, achieved by inducing reactive oxygen species (ROS) production [7]. This oxidative stress is primarily due to the re-oxidation of silver (I) ions (Ag^+^ ions) released from metallic AgNPs. The effectiveness of this antibacterial activity is highly dependent on the capping agents on the surface of the AgNPs [8]. Biological compounds such as polypeptides and proteins can be excellent capping agents, enhancing additional or selective antibacterial activities [9].

Ultraviolet (UV) radiation can have several significant impacts on biological molecules, such as peptides and proteins [10]. UV radiation can induce protein denaturation, causing the protein to lose its native structure and function [11]. High-energy UV photons can disrupt weak non-covalent bonds, such as hydrogen bonds, which stabilize the protein’s three-dimensional structure [12]. This disruption leads to the unfolding or misfolding of the protein, rendering it non-functional. UV radiation can also lead to the formation of covalent bonds between adjacent protein molecules or within the same protein molecule [13]. This process, known as crosslinking, can result in the aggregation and insolubility of proteins [14].

While having these destructive effects on proteins, UV radiation in the proper duration can alter the electrochemical condition of the AgNP’s surface so that it releases silver ions [15]. Such an alteration of the electric state of AgNPs by UV light is not surprising. Proteins generally contain numerous dipoles. The electric field of UV light can act on some of the dipoles and consequently partially oxidize the Ag atoms. Khan et al. [16] synthesized silver nanoparticles by exposing aqueous AgNO_3_ solutions, mixed with pullulan, to UV irradiation for 1 h to 96 h. Yin et al. [17] state that silver ions are more toxic to bacteria, especially Gram-negative bacteria. Mittelman et al. [18] report UV radiation on AgNPs for several days forms Ag_2_O layers, causing the release of Ag^+^ ions. They also report that long exposure to UV can induce the formation of free radicals (OH^●^) that reduces the stability (decrease in the magnitude of zeta potential) of AgNPs. If we can control the release of silver ions with UV radiation, it will open up a method to adjust cytotoxicity, possibly in a selective fashion to different bacteria.

The aim of this paper is twofold. First, we report that we have biologically synthesized stable AgNPs using CWSAE (common walkingstick (*Diapheromera femorata*) aqueous extract). Our analysis indicates the synthesized AgNPs, including the UV-treated samples, are stable for at least over 20 weeks. Second, we have found that UV-treated AgNPs can demonstrate higher selective cytotoxicity. The disk diffusion test demonstrated that CWSAE-induced AgNPs selectively inhibited the growth of the five bacteria examined after short UV radiation. UV radiation up to two minutes on the AgNPs increased the growth inhibition of *Staphylococcus aureus* USA300 and *P. aeruginosa*, whereas it showed no observable effect on *Burkholderia cenocepacia* K-56, *Klebsiella pneumoniae* ST258, or *Escherichia coli* BW25113.

We believe that the capping agents plays a role in this selective cytotoxicity. We chose those bacteria to gain insights into the selective antibacterial activity of AgNPs on different bacterial strains. The biochemical and structural variations in the bacterial cell membrane are critical factors influencing the interactions between AgNPs and bacterial cells. For example, Gram-negative bacteria such as *P. aeruginosa* and *E. coli* possess an outer membrane that may impact nanoparticle penetration and binding, while Gram-positive bacteria like *S. aureus* have a thicker peptidoglycan layer. However, our findings did not align with the simple assumption mentioned above. Therefore, it appears that not only structural and biochemical variations in bacteria play a role, but also the surface charge and structural modifications on the capping agent of AgNPs may play a crucial role in their specificity of interaction with bacterial cells.

These results validate our hypothesis that the capping agent of AgNPs is a key element for antibacterial activity. If the CWSAE has good reducing/capping agents to form and stabilize AgNPs, and UV radiation can induce structural/conformational changes in CWSAE protein-rich capping agent components (including other major capping agents such as carbohydrates), then UV-treated AgNPs can exhibit a significant change in their antibacterial activity against certain bacteria. Therefore, we propose that UV exposure time-controlled AgNP treatment/synthesis can serve as a potential foundation for developing selective antibacterial agents targeting specific bacteria. To confirm this hypothesis further, we investigated the effects of UV treatment on the selective cytotoxicity of the AgNPs, considering potential scenarios of UV influence on the protein/peptide-rich capping agents of the AgNPs.

## 2. Materials and Methods

### 2.1. The Common Walkingstick Extract (CWSAE) Preparation at Different Concentrations

One of the common walkingstick (CWS) specimens was collected in Hammond, LA, USA. One CWS (mass: 1.02 g) was added to Millipore water (100 mL) and blended (Figure A1). The mixture was centrifuged at 4000 rpm for 50 min. The supernatant was micro-filtered (pore size, 0.4 μm) for purification and to prevent possible biological contaminations. The CWSAE was frozen at −70 °C and freeze-dried into powder form at −40 °C for 30 h using the Scientific Pro Freeze Dryer from HARVESTRIGHT. The CWSAE powder was re-dissolved in Millipore water to produce AgNPs. The CWSAE concentration (in mg/mL) was also confirmed using a UV-Vis spectrophotometer (Thermo Evolution 220, Thermo Scientific, Waltham, MA, USA).

### 2.2. FTIR and BCA/Bradford Assay

An FTIR spectrometer (Thermo Scientific, Waltham, MA, USA, Nicolet iS10, Smart iTX) was employed to analyze the chemical components of the CWSAE and CWSAE-induced AgNPs. The FTIR spectrometer collected spectra in the wavenumber range of 400–4000 cm^−1^ with 16 scans. Bicinchoninic acid (PierceTM BCA Protein Assay Kit) and Bradford assays (BIO-RAD Bio-Safe Coomassie G-250 Stain, Hercules, CA, USA) were performed to detect the presence of polypeptides in CWSAE. 

Bicinchoninic acid (PierceTM BCA Protein Assay Kit) analysis was also performed for CWSAE-induced AgNPs. Each 300 µL of CWSAE, with a protein concentration of 0.297 mg/mL, was mixed with 50 µL of AgNO_3_ at various concentrations (20 mM, 50 mM, 100 mM) to create mixtures designated as CWSAE-AgNP20 for the 20 mM group, CWSAE-AgNP50 for the 50 mM group, and CWSAE-AgNP100 for the 100 mM group. The CWSAE-AgNO_3_ mixtures were then incubated at room temperature (20 °C) for ~30 h. Subsequently, the formed AgNPs were isolated by centrifugation for 30 min at 14,000 rpm, using the Labnet Spectrafuge 16M Benchtop Micro-Centrifuge (Edison, NJ, USA). Bicinchoninic acid (BCA) assays were performed, with three repetitions for each, to measure the protein concentration in CWSAE samples after the isolation of the AgNPs.

### 2.3. Synthesis of CWSAE-Induced AgNPs

AgNPs were synthesized by mixing 700 µL of CWSAE (1.0%, mass/volume percent, (*m*/*v*)) and 100 µL of Ag^+^ ion solution (AgNO_3_, 100 mM). AgNPs formed within 24–48 h at room temperature (approximately 20 °C). The aqueous CWSAE-induced AgNP solution was diluted with distilled water to a total volume of 10 mL and stored at 4.0 °C for later use.

AgNP samples were synthesized all at once and divided into three groups. The first group (called the 4-week-stored group) was stored at 4 °C for 4 weeks after the synthesis and prior to UV treatment. Within the following 48 h, inhibition tests (see below) were conducted, followed by UV-Vis spectroscopic measurements. The second group (called the 20-week-stored group) was stored at 4 °C for another 16 weeks (20 weeks in total) before UV treatment followed by UV-Vis spectroscopic measurement within one hour. The third group (called the room-temperature-stored group) was left at room temperature since the first spectroscopic study and before another spectroscopic study (16 weeks left at room temperature before the second spectroscopic study). 

### 2.4. Characterization of the CWSAE-Synthesized AgNP with UV Treatment

The diluted CWSAE-induced AgNP solution was divided into six groups (depending on UV exposure durations) of three hundred-microliter (300 µL) samples. Each of these samples was exposed to UV light with a wavelength of 302 nm at the low intensity setting for various durations (1 min, 2 min, 5 min, 10 min, and 30 min) on a UV Transilluminator (Model: TFM-30, 4 × 25 W/230 V/50 Hz/2.0 Amp). We will call these samples 0-min group, 1-min group, 2-min group, 5-min group, 10-min group, and 30-min group, respectively. 

The size and other properties of AgNPs were characterized using a UV-Vis spectrometer (Thermo Evolution 220, Thermo Scientific, Waltham, MA, USA). Transmission electron microscopy (TEM) with a JEOL 2010 instrument (200 kV) (Tokyo, Japan) and scanning electron microscopy (SEM) with Energy-Dispersive X-ray Spectroscopy (EDS) using an FEI Quanta 650 FEG (Thermo Scientific, Waltham, MA, USA) were also employed for imaging the AgNPs. TEM imaging was used to evaluate the overall dimension of AgNPs, and SEM imaging was used to characterize the degree of aggregation of AgNPs.

### 2.5. Disk Diffusion Test of (UV-Treated) CWSAE-Induced AgNPs

Fifty µL of Luria Broth (LB)-cultured bacteria was applied to Mueller–Hinton agar and evenly spread using a sterile T-shaped spreader. Next, Whatman AA disks with a 6 mm diameter were placed onto the inoculated agar surface. Subsequently, 18 µL of the AgNP solution from each group was added to the respective disks, and the agar plates were incubated at 37 °C. The diameters of the inhibition zones were (3 repetitions each sample) measured in millimeters after a one-day incubation period.

## 3. Results and Discussion

### 3.1. CWSAE Component Analysis

#### 3.1.1. FTIR (Fourier Transform Infrared) Spectral Analysis

**Figure 1:**
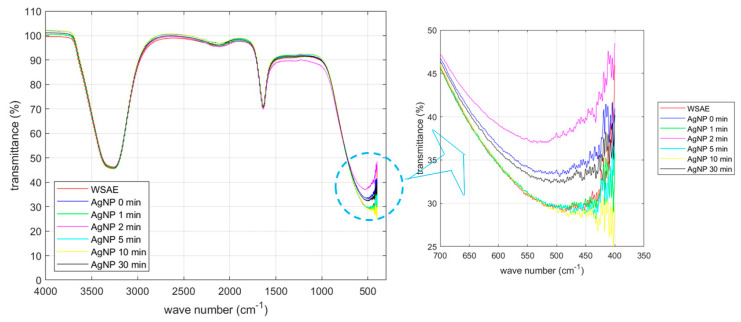
FTIR of CWSAE and UV-treated CWSAE-induced AgNPs: There were two main peaks, indicating the NH/OH band and the C=O band (with water bending). These peaks suggest that the CWSAE and the capping agents of AgNPs are primarily composed of polypeptides and other water-soluble biological molecules, such as carbohydrates, phenols, alkaloids, and organic acids with water molecules.

The CWSAE solution is a mixture of water-soluble compounds extracted from the common walkingstick. Figure 1 shows the FTIR (Fourier Transform Infrared) transmission spectrum (called the IR spectrum, hereafter) obtained in this study for the CWSAE, the CWSAE-synthesized AgNP without UV radiation, and the CWSAE-synthesized AgNP with UV radiation of various durations. Two main valleys observed near 3300 cm^−1^ and 1750 cm^−1^ can be identified as absorptions by NH/OH (the stretching vibration) and by C=O (the amide I band) plus H-O-H (the bending vibration of water), respectively.

The IR spectrum of the CWSAE appears to display characteristic absorptions associated with the aqueous protein extract solution [19,20]. However, the FTIR data of the extract sample may exhibit a limited capability in revealing its composition due to various factors. Extracts often consist of a diverse array of compounds with distinct chemical structures, making it challenging to discern individual components accurately using FTIR alone. The presence of overlapping absorption bands further complicates the identification process, as compounds with similar functional groups may contribute to blended signals. Additionally, our tested CWSAE might be at too low concentrations (≤1.0%) of certain components, resulting in weak signals that are challenging to detect amidst more abundant constituents. Therefore, these spectroscopic interpretations were further validated with the BCA (Bicinchoninic acid) and Bradford assays. The results indicate that protein-based biomolecules, including amino acids, peptides, and proteins, constitute ≥ 42.5% of the mass percentage of the biological components of the CWSAE. 

The IR spectrum of the CWSAE-synthesized AgNP without UV radiation appears similar to that of the CWSAE; the two spectral lines are nearly identical except for the valley near 500 cm^−1^. The spectral valley near 500 cm^−1^ draws particular interest. The significant difference in the peak transmission between the WSAE-only and AgNP without UV radiation cases (~29% vs. 33%) indicates that the reduction process responsible for AgNP formation involves dynamics in the frequency range of 500 cm^−1^ (15 THz). This frequency range is associated with photo absorption by large molecules [21,22]. In the present case, it is likely that the dynamics of the secondary structure of the protein play an important role in the formation of AgNP. 

#### 3.1.2. Protein Concentration Measurement for Three Mixture Groups

**Figure 2:**
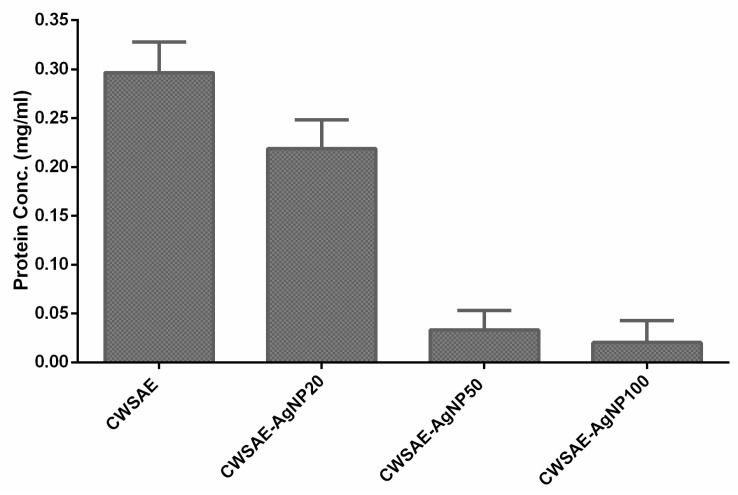
Decrease in CWSAE protein concentration after isolation of the formed AgNP in the CWSAE: The measured protein concentrations of CWSAE decreased further when a higher quantity of the formed AgNPs was isolated in CWSAE. This suggests that the capping agent of the formed AgNPs contains proteins from CWSAE.

To confirm that the CWS proteins play a significant role in forming AgNPs by reducing the silver ions in the solution, we measured the remaining proteins for the three groups (CWSAE-AgNP20, CWSAE-AgNP50, and CWSAE-AgNP100) mentioned above. Figure 2 compares the protein concentration in the solution after the isolation of AgNPs with the original concentration. It shows that the remaining protein concentration decreases as the added silver ion concentration increases. This result indicates that CWSAE proteins play a significant role in the bioreduction process of precursor Ag^+^ ions, leading to the formation of AgNPs. It also indicates that the proteins contribute to the composition of the capping agent of the resulting AgNPs. 

#### 3.1.3. Effect of UV Radiation on AgNP Formation

Furthermore, Figure 1 exhibits that the changes in the peak transmission due to UV radiation with various exposure times are similar to the above change caused by mixing CWSAE into the AgNO_3_ solution. For instance, after 1 min of UV radiation, the peak transmission goes down to ~29%, whereas after 2 min radiation, it jumps to ~37%. These are approximately 4% swings around the 0-min radiation case. Other radiation durations cause the same level of changes in peak transmission. This similarity indicates the possibility that UV radiation affects the same dynamics as the reduction process. 

This observation leads to the possibility that UV radiation changes the way the protein molecules are attached to the AgNP. It is interesting to note that the central wavelength of UV A light (302 nm) is 993 THz in frequency. The secondary structure associated with 15 THz is not susceptible to this frequency range (993 THz is too fast for the secondary structure to respond). This interpretation leads to the following speculation regarding the effect of UV radiation. UV light is absorbed by the primary structure of short peptides and/or primary structure-like portions of loops between secondary structures [15], and the absorbed energy is somehow transferred to the adjacent secondary structures of proteins. This speculation is consistent with our previous study showing that UV radiation likely affects the disulfide bonds of the BSA (Bovine Serum Albumin) molecule. 

### 3.2. Characterization of AgNP

#### 3.2.1. Visual Examination

**Figure 3:**
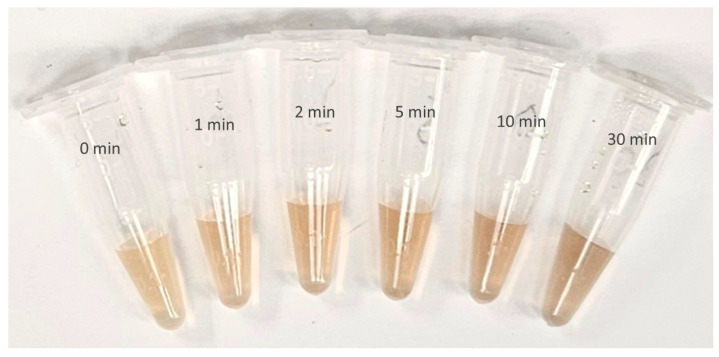
AgNP formation from CWSAE-Ag^+^ ion complex after different UV exposure time treatments (from left to right, 0 min, 1 min, 2 min, 5 min, 10 min, and 30 min): Ag^+^ ions were successfully reduced in metallic form in CWSAE in aqueous solution, showing a yellowish-brown color within 24–48 h. AgNPs exposed to UV for ten and thirty minutes exhibit significant aggregation.

Figure 3 shows the CWSAE-induced AgNP formed in the present study. Ag^+^ ions exhibited signs of reduction into metallic form when mixed into CWSAE, showing a yellowish-brown color as depicted in Figure 3. Note that AgNP samples exposed to UV for 10 and 30 min exhibit significant aggregation.

The overall surface net charge of CWSAE proteins (and/or polypeptides) tends to be negative due to the presence of negatively charged amino acid residues. Additionally, certain free amino acids present in the CWSAE, originating from the hemolymph of the common walkingstick (CWS), may contribute to the formation of AgNPs. The negative charges of aspartic acids and glutamic acids in the CWS hemolymph, found in the CWSAE, may also facilitate the reduction process of Ag^+^ ions within the aggregates.

#### 3.2.2. TEM Analysis

**Figure 4:**
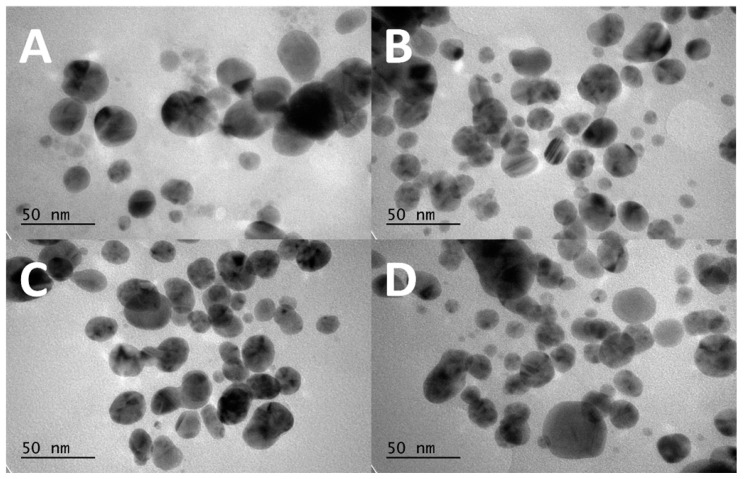
TEM images of AgNPs obtained at different UV exposure times: (**A**) control group of the CWSAE-induced AgNPs without any UV exposure time, (**B**) the CWSAE-induced AgNPs with 1 min of UV exposure time, (**C**) the CWSAE-induced AgNPs with 2 min of UV exposure time, and (**D**) the CWSAE-induced AgNPs with 30 min of UV exposure time.

Figure 4 shows TEM images of the AgNP samples with various UV exposure times. There is no obvious change in the dimensions or shape of the particles caused by the UV radiation. The average dimension of the particles can be estimated in the vicinity of 50 nm. The only slight change observed in this figure is that, with 30 min radiation, the AgNPs is somewhat elongated. This observation is consistent with the argument that the AgNPs can turn elliptical in association with mode-splitting due to UV radiation. (See below for the mode-splitting argument).

### 3.3. UV-Vis Spectroscopic Analysis

The surface area-to-volume ratio of a sphere decreases as its radius increases. Therefore, as the size increases, the AgNP’s interaction with bacteria, hence its cytotoxicity, becomes less effective. It is important to clarify the meaning of the word ‘size’ used in this paper. There are two main factors that affect the cytotoxicity of AgNP. The first is the size of the silver particle itself. It is determined by the reduction process induced by the capping agent. The second is the degree of aggregation. Silver nanoparticles produced by the reduction process tend to aggregate, increasing the surface area. The capping agent supposedly prevents aggregation. However, depending on its chemical structure and other factors, aggregations occur. Throughout this paper, the word ‘size’ is used to mean the size (the radius or diameter) of the core of the AgNP (metal) and ‘dimension’ to describe the overall shape of the AgNP, including the degree of aggregation. 

UV-Vis spectroscopy is based on plasmon resonance, which occurs via interaction between the spectrometer’s light source (UV-Vis) and free-electrons in the Ag particle (metal). The resonant frequency depends on the distance between the electrons and the positive charges that cause the oscillation. Therefore, the spectroscopic data, i.e., the peak wavelength, represents the size of the metal (Ag). TEM imaging offers 2D (two-dimensional) imagery at a high resolution via the analysis of electrons transmitted though the sample. When tagged with metal, TEM increases sensitivity to visualize proteins. Thus, in the present study, TEM images provide us with the overall shape of the AgNP, and we can estimate the average dimension of AgNPs. On the other hand, SEM produces an image by detecting secondary or backscattered electrons. Hence, SEM imaging offers 3D (three-dimensional) surface mapping and is therefore superior to UV-Vis spectroscopy or TEM for probing the state of aggregation.

Bohren and Huffman ([23], p. 323) discuss that, when the particle size is much smaller than the wavelength of the electromagnetic wave, absorption dominates over scattering. Thus, from the obtained transmission UV-Vis spectrum, we can derive an absorption spectrum and use it to probe the plasmon resonance. Here, the peak absorption frequency corresponds to the plasmon resonant frequency.

**Figure 5:**
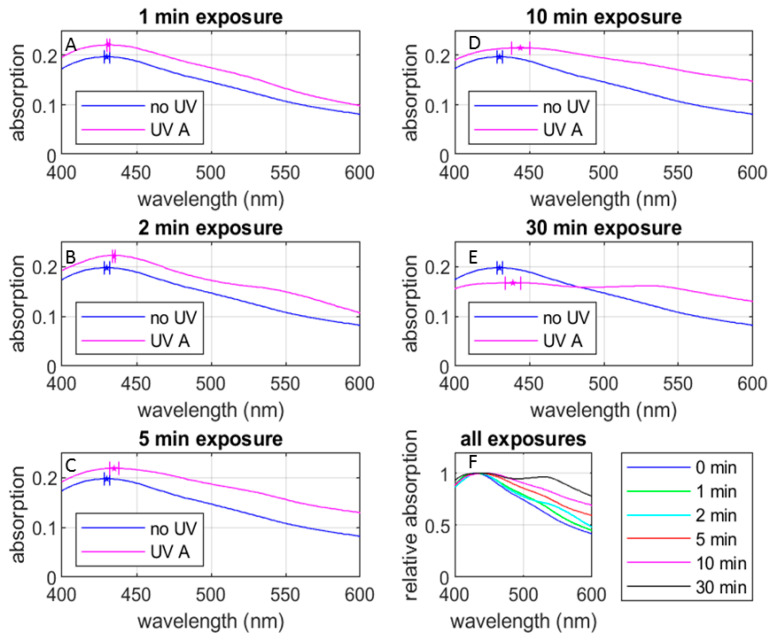
UV-Vis absorption spectra under various UV exposure durations compared with the non UV-exposed case: (**A**) 1 min UV exposure, (**B**) 2 min UV exposure, (**C**) 5 min UV exposure, (**D**) 10 min UV exposure, (**E**) 30 min UV exposure, and (**F**) relative absorption-spectra for all UV exposures. The spectral lines are from the data with 4-week-stored AgNP samples while the error bars are from the combination of 4-week-stored and 20-week-stored AgNP samples. See below for the definitions of the 4-week- and 20-week-stored samples.

**Figure A2:**
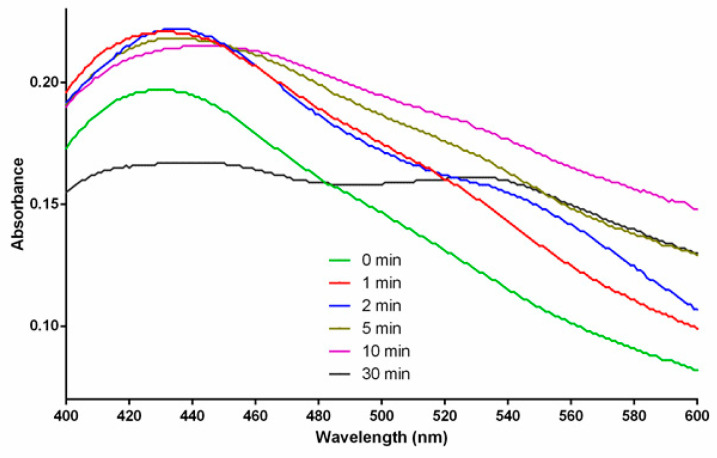
UV-Vis spectra of the UV-treated AgNPs, at different UV-Exposure Time Treatment (0 min, 1 min, 2 min, 5 min, 10 min, and 30 min.

Figure 5 shows the UV-Vis absorption spectra of the AgNP samples with various UV exposure durations (all the six groups defined in Section 2.4). It reveals that most of the spectra exhibit a consistent pattern, with an exception observed in the 30 min group. (See below for the discussion of the spectrum for 30 min of radiation). Here, Figure 5A–E compare the change in the spectral shape after the respective UV exposure durations against the spectral shape prior to the UV exposure. In each of these figures, the spectral peaks are indicated with pentagonal marks. Figure 5F compares the spectral shapes for all the UV exposure durations, including the one prior to the UV exposure. It is clearly seen that the UV exposure (1) shifts the spectral peak to the red side and (2) increases the peak absorbance, except for the exposure duration of 30 min (Figure A2 shows an expanded version of Figure 5F for better visibility), and broadens the spectral width. These tendencies are consistent with observations by Mittelman et al. [18]. Table 1 summarizes these observed changes. We discuss these observations in the following paragraphs.

Based on Mie’s theory and plasmon resonance, we can estimate the average particle size from the spectral peak frequency [23,24,25]. In the case of the 0-min and 1-min groups, the spectral peaks were centered near 430 nm, suggesting an average size of approximately 50 nm ([24], (p. 108, Figure 3)) for the formed AgNPs. For the 2-min and 5-min groups, the spectral peaks are blue-shifted to around 438 nm, indicating an average size of approximately 55 nm. In the 10-min group, the spectral peak was around 450 nm, suggesting an average size of approximately 60 nm for the formed AgNPs. 

While the spectral peak wavelength allows us to roughly estimate the core size of the AgNPs, the effect of UV radiation seems more complicated. It is challenging to discern a significant change in the dimension of the AgNPs in TEM images (Figure 4). Although the particle sizes seen in the images (the darker part of the image exhibiting the metal part of the AgNP) fall in the range shown in Table 1, Mie’s theory cannot explain the change in absorbance due to the UV radiation observed in Figure 5 and Table 1. The absorption and scattering of light by a nanoparticle depend on its chemical composition, size, shape, surrounding dielectric medium, and the coupling of the colloids and adsorbed solutes ([24], (pp. 105–106)). It is important to consider various factors in discussing the effect of UV radiation observed in the AgNP’s spectroscopic characteristics and associated cytotoxicity to bacteria. In the following sections, we discuss our UV-Vis absorption spectral data from various angles. 

#### 3.3.1. Electromagnetic Effect of UV Radiation on AgNP

To explain the fundamental interaction between UV light and proteins, here are some possible influences of UV on proteins. Electrons in the conduction band of nanoparticles interact with incoming electromagnetic waves. The oscillating electric field of the electromagnetic wave causes a collective excitation of the conduction electrons. The displacement of the electrons against the immobile positive charges, due to their much higher mass as compared with electrons, leads to the polarization of the nanoparticle. When the oscillation frequency matches the frequency of the electromagnetic wave, the phenomenon called surface plasmon resonance occurs and the corresponding energy of the electromagnetic wave is absorbed. 

Considering the UV frequency, it is likely that UV light is absorbed by the primary structure, such as short peptides and/or primary structure-like portions of loops between secondary structures, of the capping agent, as discussed above. The absorbed energy is then transferred to the adjacent secondary structures of proteins.

#### 3.3.2. Aggregation of AgNP

**Figure A3:**
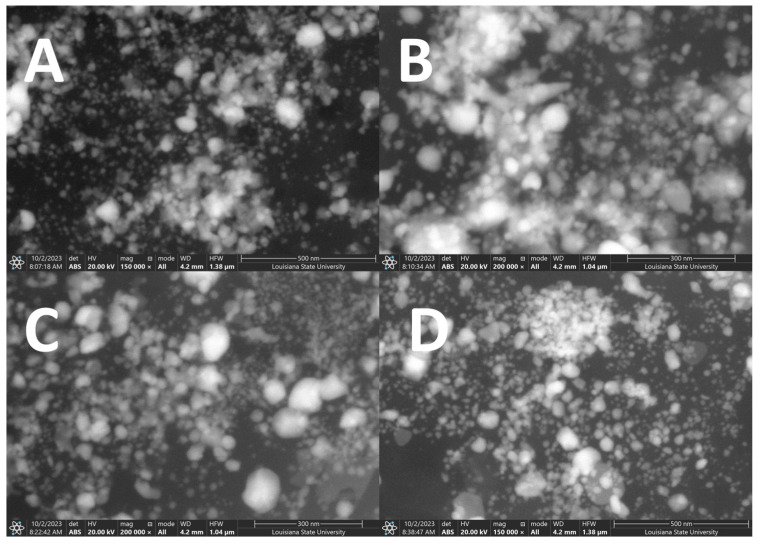
SEM images of UV-treated/CWSAE-induced AgNPs at different UV exposure times ((**A**) 0 min, (**B**) 1 min, (**C**) 2 min, and (**D**) 30 min).

The widely accepted notion is that cytotoxicity increases with a reduction in the size of nanoparticles, and capping agents play a crucial role in maintaining small particle size by preventing the aggregation of nanoparticles. In the SEM (scanning electron microscopy) images (Figure A3), all our sample groups (1 min, 2 min, 5 min, 10 min, and 30 min UV-treated AgNP groups) exhibit signs of aggregation. As mentioned above, SEM imaging is superior to TEM or UV-Vis spectroscopy for detecting aggregations as it can be used to estimate the 3D structure of nanoparticle samples.

**Figure A4:**
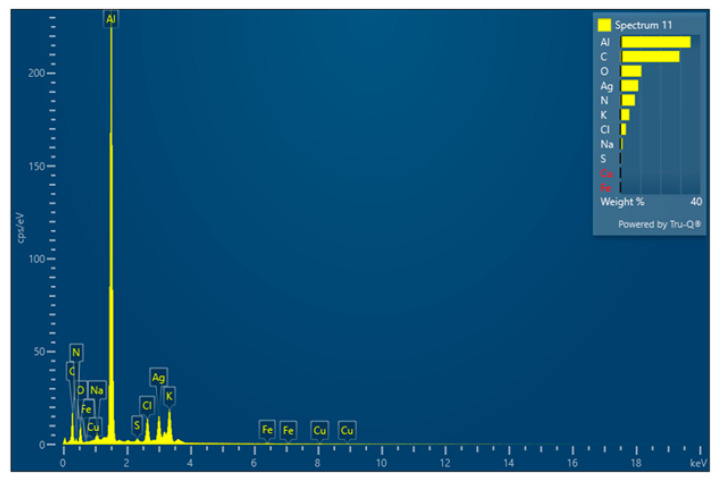
EDS Data of the Synthesized AgNPs.

Thus, the observed alterations in the maximum peak wavelength and absorbance of the UV-Vis spectra of the AgNPs could be attributed to their increased aggregation induced by UV radiation. As mentioned above, Mittelman et al. [18] report aggregation by UV radiation and decrease in the magnitude of the zeta potential by UV radiation. The aggregation of AgNPs is believed to result from protein–protein interactions among the protein-based capping agents on the surface of biologically reduced AgNPs (Figure A4). However, in the groups subjected to longer UV treatment times (10 min and 30 min), we observed a greater amount of precipitated, aggregated AgNPs. 

At this time, we cannot explain the mechanism of the observed UV-induced aggregation of AgNP samples. However, based on the abovementioned interpretation that the optical energy of UV light is absorbed by the primary structures and transferred to the secondary structures, the following argument may be plausible. The restructuring of the secondary structure alters the electric charge distribution over the capping agent. Consequently, the interaction between the capping agents of AgNPs changes and it leads to aggregation of the AgNPs. The exposure time is considered to be proportional to the absorbed energy, hence the degree of the change in the inter-capping agent interaction. This consideration may explain why longer UV radiation causes more aggregation. 

The dual-peak spectral shape observed in the absorption spectrum of the 30-min group may indicate the aggregation. Bohren and Huffman ([23] (p. 373, Figure 12.19)) discuss that, in plasmon resonance of AgNPs, mode splitting can occur when the nanoparticles are elongated. Here, the greater the particle elongation, the greater the separation of the two peaks, where the red-side peak is red-shifted and the blue-side peak is blue-shifted. We can see these tendencies in Table 1 by comparing the spectra of the 10-min and 30-min groups. When the radiation duration is increased from 10 min to 30 min, the peak at 444 nm observed in the 10-min group is shifted to 439 nm and a new peak appears at 528 nm. Furthermore, this new peak at 528 nm in the 30-min group can be interpreted as a slight red-shift of the faint peak observed at approximately 525 nm in the 10-min group. It is possible to interpret that aggregation causes the elongated shape of the AgNPs and that the increase of UV radiation duration from 10 min to 30 min increased the elongation. 

#### 3.3.3. Effect on AgNP Size 

**Figure 6:**
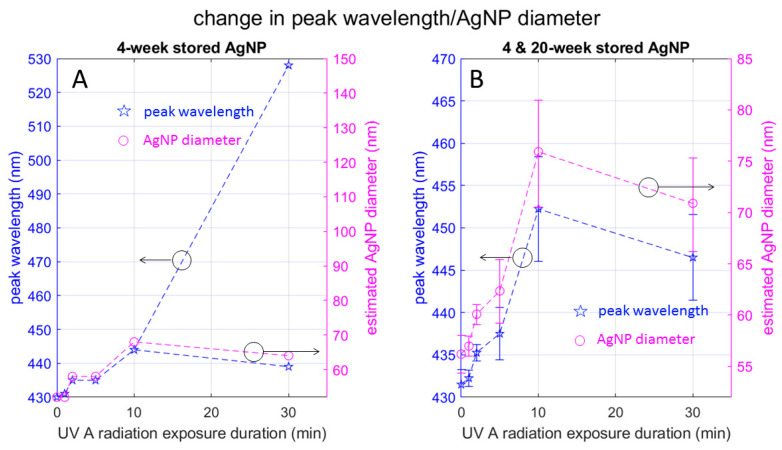
Change in UV spectrum peak wavelength and AgNP diameter with UV radiation: Plots in (**A**) depict data from 4-week-stored samples, highlighting the data point corresponding to the red-side peak observed in the 30-min exposed sample. Plots in (**B**) show a combination of 4-week- and 20-week-stored samples, with error bars. These plots elucidate the reduction in particle size observed during radiation, specifically from 10 min to 30 min.

As mentioned above, the UV-Vis absorption spectrum depends on various factors. In this section, we elaborate on the AgNP particle size estimation made in Table 1. Figure 6 plots the peak wavelength seen in Figure 5 as a function of the UV exposure duration, along with the AgNP diameter estimated based on Figure 3 of [24]. Here, Figure 6A is based on the samples in the 4-week-stored group and includes the double-peak wavelengths observed in the split spectrum for the 30-min sample group. Notice that the red-side peak wavelength 528 nm is out of the range of Figure 3 of [24] and we were unable to estimate the corresponding particle diameter. Figure 6B eliminates the red-side peak wavelength for a close-up view of Figure 6A and uses error bars based on the combination of the 4-week-stored and 20-week-stored samples. 

Figure 6A indicates that the AgNP diameter increases monotonically with the UV radiation duration if we disregard the red-side peak for the 30-min group. On the other hand, the AgNP diameter corresponding to the blue-side peak wavelength for 30 min radiation (64 nm) is smaller than the peak wavelength for the 10 min radiation (68 nm). Based on the mode-splitting discussion by Bohren and Huffman ([23] (pp. 145, 343, and 373)) we can interpret that the red-side and blue-side peak wavelengths respectively correspond to the resonant frequency of the longer and shorter diameter of the ellipse. In other words, UV radiation for 30 min elongates the AgNP so that the major axis of the resultant ellipse is longer than the diameter of the more spherical particle formed by 10 min radiation, and the minor axis is shorter. 

#### 3.3.4. Temporal Stability of AgNPs

**Figure 7:**
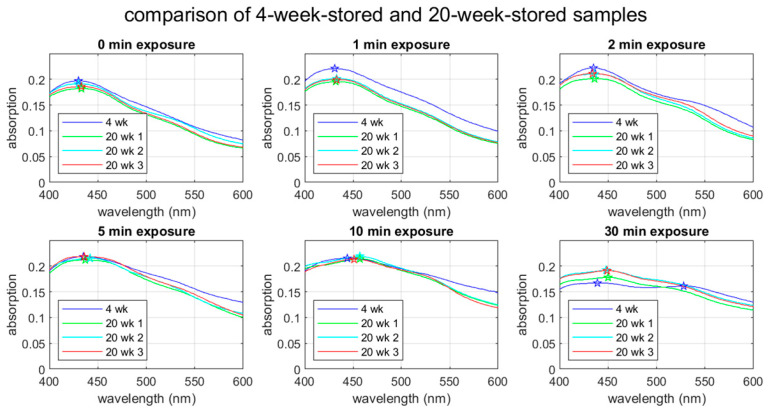
UV-Vis spectra for 4-week- and 20-week-stored samples. In the 4-week-stored case, the spectra were obtained within 48 h after the UV treatment, whereas spectra were obtained within one hour after the UV treatment for the 20-week samples. The star symbols indicate the peak wavelengths.

The stability of AgNPs over time is an important factor for their practical use. Figure 7 compares the UV-Vis spectra between the 4-week-stored and 20-week-stored groups. Note that we ran the spectroscopic measurement three times for the 20-week-stored samples to see statistical variations. The 4-week-stored data are based on a one-time spectroscopic measurement. The spectral shapes for the non-UV-treated through to the 10-min exposure cases are practically the same, showing the peak wavelength staying within ±5% of shift. The peak absorption for the 1-min exposure case appears lower in the 20-week-stored sample. The reason for this reduction in absorption is unknown. Except for this difference in the peak absorption for the 1-min exposure case, the spectral changes in 16 weeks (4 week vs. 20 weeks) seem to be within statistical variation. 

The 30-min exposure case shows a distinctive change in 16 weeks. The second (the red-side) peak in the 30-min exposure case is not seen in the 20-week-stored samples. The single peak seen in the 20-week-stored samples is located between the blue-side and red-side peaks observed in the 4-week-stored sample. While this observation is consistent with the above-discussed mode splitting due to aggregation, it is mysterious why the 4-week-storage sample shows the splitting and the 20-week-storage samples do not. It is possible that nanoparticle aggregation is a dynamic phenomenon.

**Figure A5:**
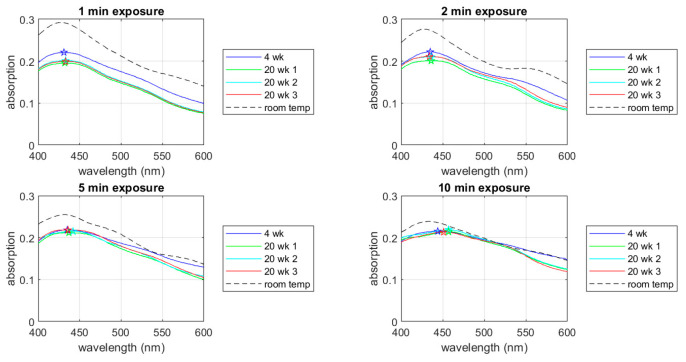
UV-Vis spectra of the room-temperature-stored samples. The star symbols indicate peak wavelengths.

The UV-Vis spectra of the room-temperature-stored samples show more or less the same features as the samples stored at 4 °C (See Figure A5). The 1-min through to 10-min groups clearly show an absorption peek, indicating their cytotoxicity is intact after storage for 16 weeks at room temperature. 

#### 3.3.5. Effect on Peak Absorbance

**Figure 8:**
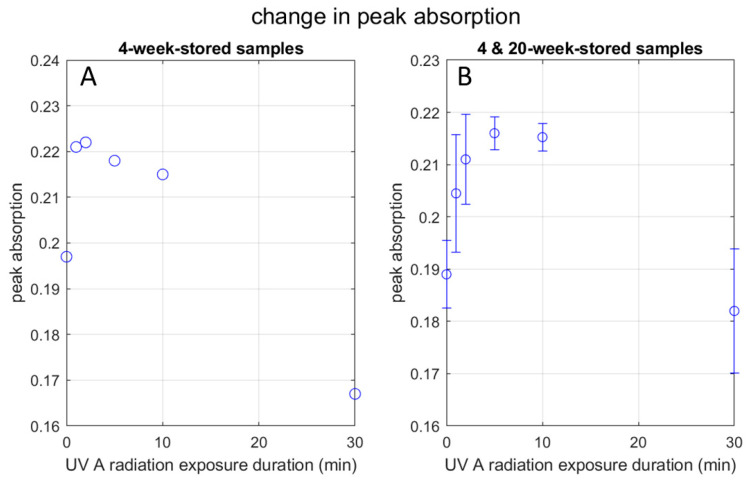
Change in UV peak absorbance with UV radiation: (**A**) graph shows the peak absorption pattern of the 4-week-stored AgNP samples at UVA radiation exposure time points in minutes; (**B**) graph shows the peak absorption pattern in combination with the 4-week-stored AgNP samples and the 20-week-stored AgNP samples together at UVA radiation exposure time points in minutes, with error bars.

Figure 8A plots the peak absorbance observed in the UV-Vis spectra of the 4-week-stored samples as a function of UV exposure duration. It increases up to 2 min of radiation and monotonically decreases afterward. As for the 30-min group, the peak absorbance for the red-side and blue-side peak wavelengths are approximately the same (0.161 vs. 0.167). Practically the same peak absorbance between the two peak wavelengths indicates that the two spectral peaks belong to the same physical entity, supporting the above thoughts regarding elongation of the AgNP.

Figure 8B is the case when we combine the 4-week-stored and 20-week-stored samples. A similar dependence of the peak absorption on the UV exposure duration as Figure 8A is seen, except that the absorption increases up to 5 min of radiation. The discrepancy of the peak absorbance UV exposure duration between 2 min and 5 min does not seem to be significant. It is likely that, in some cases, the AgNP becomes more energy-dissipative after 2 min of radiation and other time after 5 min. (See below for the discussion of energy dissipation).

Slistan-Grijalva et al. [24] report in their theoretical study based on Mie’s theory that, while the peak wavelength increases with the radius of the AgNP particle, the peak absorbance increases with the particle radius up to 18 nm (36 nm in diameter), then decreases up to the radius of 40 nm (80 nm in diameter). (Their analyses are supported by experiments.) As Table 1 indicates, the estimated diameters in the present study (except for the larger diameter observed in the 30-min group) fall in this range of 36–80 nm in diameter, where the absorbance decreases with the particle size. However, as shown by Figure 8A,B, the peak absorbance increases up to 2 min or 5 min. We need an explanation for this increase. We will discuss it in the next paragraph.

Based on the statements made by Bohren and Huffman ([23], (p. 323)) and Slistan-Grijalva et al. [24], it is likely that the increase in the absorbance observed up to 2 min of radiation can be attributed to factors other than the surface plasmon resonance, such as the change in the particle shape and the environmental dielectric condition. In particular, the following speculation is plausible. When the electric field of UV light oscillates the hydrophilic region of the capping agents, it loosens their attachment to the AgNP. Consequently, there is a chance that the capping agents move relative to the AgNP surface, causing energy dissipation (something like friction). This effect contributes to the imaginary part of the overall electric permittivity, causing the UV energy to dissipate. Lee et al. explain this effect, calling it surface damping [26]. Interestingly, this change in the possible surface condition may contribute to the toxicity to bacteria (See below). 

### 3.4. Influence of UV Treatment on Selective Cytotoxicity of CWSAE-Induced AgNPs

**Figure 9:**
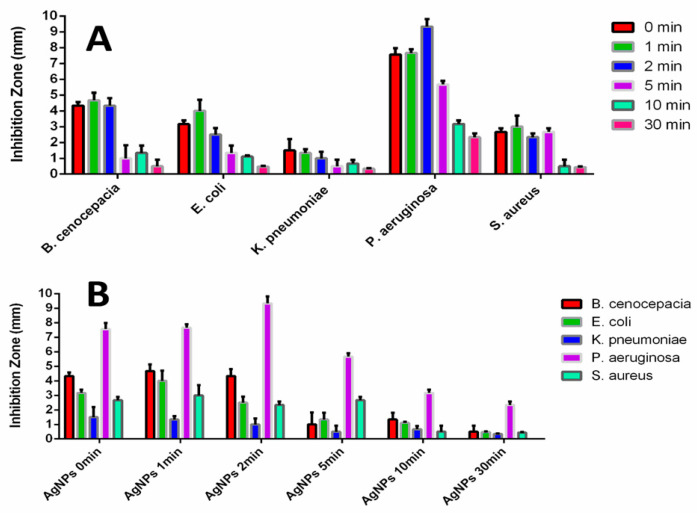
Inhibition zone graphs of UV-treated AgNPs against five bacteria: (**A**) Inhibition Zone data of different UV-Exposure time grouped by the same tested bacteria: The inhibition Zones become larger at the short UV-Exposure time (1 min and 2 min, compared to 0 min as a control group) and smaller at the long UV-Exposure times (5 min, 10 min, and 30 min) and (**B**) Inhibition Zone data of different bacteria data grouped by the same UV-Exposure time: For *P. aeruginosa*, AgNPs 2 min, AgNPs 10 min, and AgNPs 30 min groups show relatively higher inhibition zones than the ones of the other bacteria. For *S. aureus*, AgNPs 5 min groups show relatively higher inhibition zones than the ones of the other bacteria except P. aeruginosa. (The inhibition zones were measured as the diameter of the inhibition zone in millimeters minus 6 millimeters (the diameter of the disk)).

Figure 9 shows the inhibition zone observed in our disk diffusion test under various conditions. The inhibition zones of the AgNPs on the disks increased with shorter UV exposure times (1 min and/or 2 min) compared to the control group (0 min) but decreased at longer UV exposure times (5 min, 10 min, and 30 min) against all five bacteria. Specifically, the AgNPs treated with UV for 2 min exhibited the largest inhibition zones against *P. aeruginosa* among the five bacteria (Figure 9A), while those treated with UV for 5 min showed higher inhibition zones against *S. aureus* than the others except for *P. aeruginosa* (Figure 9B). 

**Figure A6:**
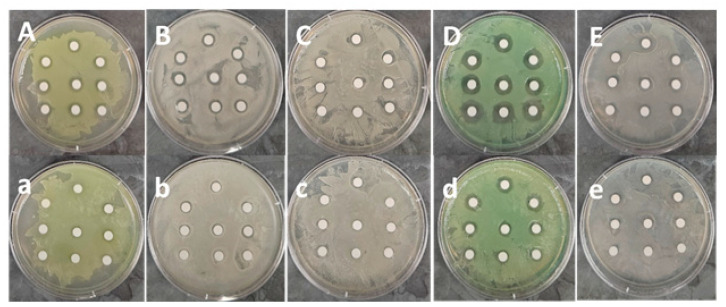
Inhibition Zone of UV-Treated AgNPs in Disk Diffusion Test: (**A**–**E**) There are 3 repetitions for 3 groups 0 min (top 3 discs), 1 min (middle 3 discs), and 2 min groups (bottom 3 discs) (**a**–**e**) There are 3 repetitions for the other 3 groups: 5 min (top 3 discs), 10 min (middle 3 discs), and 30 min groups (bottom 3 discs).

The UV-induced changes to the protein-rich components of the AgNP capping agent caused a significant difference in the cytotoxicity of the AgNPs against pathogenic bacteria. Specifically, the AgNPs treated with UV for 2 min exhibited more pronounced selective cytotoxicity against *P. aeruginosa* (Figure A6). Additionally, the AgNPs treated with UV for 5 min showed good selective antibacterial activity, not only against *P. aeruginosa* but also against *S. aureus*. These results strongly support the idea that the UV exposure time-controlled method can be a great means to induce higher selective cytotoxicity of the formed AgNPs against certain bacteria.

The selective cytotoxicity of CWSAE-induced AgNPs under UV influence can be attributed to potential alterations that occur during UV exposure. The major constituents of the capping agents for AgNPs are proteins and polypeptides, and these agents can play a pivotal role in conferring higher selective antibacterial activity to the AgNPs in controlling the UV exposure time. If UV radiation were to impact the proteins within the capping agent, it could lead to variations in cytotoxicity against pathogenic bacteria. 

The selective toxicity to bacteria is the most intriguing outcome of the present research. Although we do not have a clear explanation regarding why UV radiation increases the toxicity to *Pseudomonas aeruginosa* more than the other bacteria tested or why the toxicity of the AgNP increases up to two minutes of UV treatment, it is worthwhile speculating a possible cause (scenario) behind this selectivity. 

As Yin et al. [17] discuss, the exact mechanism of silver nanoparticles’ antibacterial effects has not been entirely clarified. However, it is known that the ionic state of silver is more toxic to bacteria than the silver atom. When an AgNP is exposed to UV light, the electric field can weaken the bond between the silver atoms and the capping agents, and consequently the polarization increases near the surface, i.e., the metallic Ag atoms become more electrically positive. These positively charged ion-like silver atoms can be attracted by the negative bacterial cell membrane potential. Yin et al. [17] discuss that, due to this electrostatic attraction and affinity to sulfur proteins, silver ions can adhere to the cell wall and cytoplasmic membrane. At this time, this change in the ionic state of silver is our hypothesis. However, it at least qualitatively agrees with ref. [18], in which Mittelman et al. report an increase of Ag^+^ release by UV radiation.

Additionally, the capping agent of the AgNPs appears to successfully maintain the cohesion between the metal nanoparticles and silver ions in the AgNPs after short-term UV light treatment. The release of already-oxidized Ag^+^ ions inside (or near) the bacterial cell body proves to be significantly more effective in targeting and killing the bacteria. This is because the capping agent of the AgNPs often serves the purpose of selectively targeting the bacteria due to their stronger interaction with certain target bacteria. However, the capping agent of AgNPs cannot protect the AgNPs over long-term UV exposure, leading to the release of oxidized Ag^+^ ions as free ions in the system (no targeting action). This is less threatening to the target bacteria compared to the ions released inside or near them. 

We believe that this explanation aligns well with our experimental results. This selective cytotoxicity of UV-treated AgNPs to bacteria may be attributed to the distinct impact of UV light on proteins compared to one of the common insect carbohydrates (like chitin, glycogen and glucose) within the capping agents. The speculation that proteins are responsible for selective cytotoxicity arises from the differential response of proteins and other capping agents such as carbohydrates (abundant in insects) to UV light, influenced by their unique chemical structures and compositions [27]. For example, chitin, the most common polysaccharide composed of N-acetylglucosamine units in insects, usually exhibits higher stability and is less susceptible to UV-induced changes. The presence of β-1,4 glycosidic linkages in chitin creates a robust and extended structure, limiting its absorption of UV light and reducing the likelihood of significant structural alterations. Proteins, being more sensitive to UV light, undergo conformational changes, peptide bond breakage, and crosslinking reactions upon exposure, resulting in significant structural alterations. Therefore, the selective cytotoxicity observed in certain bacteria may be linked to the enhanced sensitivity of proteins to UV light, causing more pronounced structural changes compared to the relatively stable and UV-resistant nature of chitin. 

Another possibility is that carbohydrates in the capping agent play a role in the UV-enhancement of cytotoxicity. As mentioned above, Khan et al. [16] report that UV radiation enhances the reduction of Ag^+^ ions by pullulan. However, their study indicates that the effect on reduction keeps increasing up to 96 h. In our case, the inhibition effect decreases after 2 min of radiation. In addition, the content of pullulan is not significant in our CWSAE sample. Therefore, we believe that proteins play a significant role in cytotoxicity. 

The decrease in toxicity, as observed by the reduction in the inhibition zone in Figure 9 after 2 min of exposure, can be attributed to changes in surface conditions compromising the interaction between bacteria and AgNP. The aggregation due to UV radiation and corresponding changes in shape likely contribute to the reduction in cytotoxicity. Judging from Figure 6, the change in Ag (i.e., the metal) particle size and shape seem to be minimal for exposure times up to 5 min. Prolonged UV exposure may also destabilize the AgNPs, leading to the release of free Ag^+^ ions, and the AgNPs become less likely to target bacteria, resulting in lower cytotoxicity.

## 4. Conclusions

Silver nanoparticles were successfully synthesized using an aqueous extract from the common walkingstick. The UV exposure time of the UV-treated AgNPs has been found to play a significant role in controlling selective antibacterial activity against certain pathogenic bacteria. We suspect that the best enhancement of cytotoxicity to these bacteria with two-min UV treatment is related to the change in the ionic state of AgNPs, whereas the selective cytotoxicity is related to the structural change of the capping agent components. These arguments must be examined further and are the most prioritized subject of our future investigation. 

Furthermore, as a green biochemical synthesis method, the synthesis of AgNPs induced by CWSAE also holds grand promise for treating pathogenic infectious diseases for humans. This approach offers advantages in medical fields, including ease of production, safety, cost-efficiency, selectivity in antibacterial properties, and environmentally friendly production, thus minimizing potential side effects.

## Figures and Tables

**Table 1:** Observed changes in peak absorption and peak wavelength by UV radiation. Absorbance is normalized to the non-exposed case.

UV exposure (min)	0	1	2	5	10	30	30
Peak wavelength (nm)	430	431	435	435	444	439	528
Peak absorbance (r.u.)	1.00	1.12	1.12	1.10	1.09	0.85	0.82
Estimated AgNP diameter (nm)	52	52	58	58	68	64	-

Note: AgNP diameters were estimated from Figure 3 of Slistan-Grijalva [24].

## Data Availability

Data are contained within the article.

## Appendix A

The body structure of the captured CWS is well-characterized, featuring a lean and elongated body with long and thin antennae, resembling a twig or branch. This structure facilitates effective camouflage in its natural environment. The coloration of the CWS typically mimics the plants surrounding it, ranging from various shades of brown to green, or featuring mottled patterns for enhanced concealment.

Most of the spectra still exhibit a similar pattern, except for the spectrum at 30 min. The highest broad peaks in 0 min and 1 min spectra are located around 430 nm, indicating the size of the formed AgNPs at an average size of approximately 52 nm. The highest broad peaks in 2 min and 5 min spectra are located around 435 nm, indicating the size of the formed AgNPs at an average size of approximately 58 nm. The highest broad peak in 10 min spectrum is located around 444 nm, indicating the size of the formed AgNPs at an average size of approximately 68 nm. Two peaks appearing in the 30 min spectrum indicate a mode splitting causing elongation of the particle ([23], (p. 373, Figure 12.19)).

SEM images predominantly reveal aggregate forms among the generated AgNPs. The dimension and shape of the AgNPs formed did not exhibit high uniformity across all UV-exposure treatments. Changes in dimension and shape among the AgNPs in the four groups may be attributed to variations in protein structures resulting from different UV-Exposure Times. In general, SEM is superior in getting surface information to TEM. Thus, in our case, SEM is a better option to investigate the degree of aggregation than TEM.

The silver nanoparticles (AgNPs) were both synthesized and capped with biological molecules, incorporating elements such as C, N, and O. This suggests that the biological molecules commonly found in the common walkingstick (CWS) body include proteins, polypeptides, and other typical compounds containing nitrogen and oxygen. Additionally, an aluminum signal was detected, attributed to the AgNPs being placed on aluminum tape.

The room-temperature-stored samples were UV-treated 16 weeks after the AgNPs were synthesized. During this period, the AgNP samples were stored at room temperature. The UV-Vis spectra show higher absorption peak than the 4-week-stored or the 20-week-stored samples. The higher absorption likely indicates higher energy dissipation due to AgNP aggregations or other damping mechanism on the nanoparticle surface. It should be noted that each of the UV-treated samples shows a clear absorption peak indicating the free electrons in the Ag particle still resonate with the UV-light of the spectrometer. This observation indicates that the AgNP keeps the cytotoxicity when stored at room temperature for 16 weeks. Also, it is interesting to note that the 2-min exposed sample in this case show double peaks where the blue-side peak wavelength is shorter and the red-side peak wavelength is longer than the 4 °C stored samples. This feature is consistent with the mode-splitting discussed in Section 3.3.4.

There are five bacteria tested for the disk diffusion test: (A/a) *Burkholderia cenocepacia* K-56, (B/b) *Escherichia coli* BW25113, (C/c) *Klebsiella pneumoniae* ST258, (D/d) *Pseudomonas aeruginosa* PAO1, (E/e) *Staphylococcus aureus* USA300 (A/a–E/e) The observed inhibition zone in our disk diffusion test varied under different UV-Exposure time conditions. The inhibition zones of the silver nanoparticles (AgNPs) on the discs increased when exposed to shorter UV times (1 min and/or 2 min) compared to the control group (0 min), but decreased with longer UV exposure times (5 min, 10 min, and 30 min) against all four pathogenic bacteria and *E. coli*.
